# Raman Metabolomics of *Candida auris* Clades: Profiling and Barcode Identification

**DOI:** 10.3390/ijms231911736

**Published:** 2022-10-03

**Authors:** Giuseppe Pezzotti, Miyuki Kobara, Tamaki Nakaya, Hayata Imamura, Tomoya Fujii, Nao Miyamoto, Tetsuya Adachi, Toshiro Yamamoto, Narisato Kanamura, Eriko Ohgitani, Elia Marin, Wenliang Zhu, Toshihisa Kawai, Osam Mazda, Tetsuo Nakata, Koichi Makimura

**Affiliations:** 1 Ceramic Physics Laboratory, Kyoto Institute of Technology, Sakyo-ku, Matsugasaki, Kyoto 606-8585, Japan; 2 Department of Immunology, Graduate School of Medical Science, Kyoto Prefectural University of Medicine, Kamigyo-ku, 465 Kajii-cho, Kyoto 602-8566, Japan; 3 Department of Orthopedic Surgery, Tokyo Medical University, 6-7-1 Nishi-Shinjuku, Shinjuku-ku, Tokyo 160-0023, Japan; 4 Department of Dental Medicine, Graduate School of Medical Science, Kyoto Prefectural University of Medicine, Kamigyo-ku, Kyoto 602-8566, Japan; 5 Biomedical Research Center, Kyoto Institute of Technology, Sakyo-ku, Matsugasaki, Kyoto 606-8585, Japan; 6 Division of Pathological Science, Department of Clinical Pharmacology, Kyoto Pharmaceutical University, 5 Misasagi Nakauchi-cho, Yamashina-ku, Kyoto 607-8414, Japan; 7 Department of Oral Science and Translational Research, College of Dental Medicine, Nova Southeastern University, 3301 College Ave, Fort Lauderdale, FL 33314, USA; 8 Medical Mycology, Graduate School of Medicine, Teikyo University, Itabashi-ku, Tokyo 173-8605, Japan

**Keywords:** *Candida auris* clades, Raman spectroscopy, metabolomics, profiling, Raman barcodes

## Abstract

This study targets on-site/real-time taxonomic identification and metabolic profiling of seven different *Candida auris* clades/subclades by means of Raman spectroscopy and imaging. Representative Raman spectra from different *Candida auris* samples were systematically deconvoluted by means of a customized machine-learning algorithm linked to a Raman database in order to decode structural differences at the molecular scale. Raman analyses of metabolites revealed clear differences in cell walls and membrane structure among clades/subclades. Such differences are key in maintaining the integrity and physical strength of the cell walls in the dynamic response to external stress and drugs. It was found that *Candida* cells use the glucan structure of the extracellular matrix, the degree of α-chitin crystallinity, and the concentration of hydrogen bonds between its antiparallel chains to tailor cell walls’ flexibility. Besides being an effective ploy in survivorship by providing stiff shields in the α–1,3–glucan polymorph, the α–1,3–glycosidic linkages are also water-insoluble, thus forming a rigid and hydrophobic scaffold surrounded by a matrix of pliable and hydrated β–glucans. Raman analysis revealed a variety of strategies by different clades to balance stiffness, hydrophobicity, and impermeability in their cell walls. The selected strategies lead to differences in resistance toward specific environmental stresses of cationic/osmotic, oxidative, and nitrosative origins. A statistical validation based on principal component analysis was found only partially capable of distinguishing among Raman spectra of clades and subclades. Raman barcoding based on an algorithm converting spectrally deconvoluted Raman sub-bands into barcodes allowed for circumventing any speciation deficiency. Empowered by barcoding bioinformatics, Raman analyses, which are fast and require no sample preparation, allow on-site speciation and real-time selection of appropriate treatments.

## 1. Introduction

In the past decade, *Candida auris* (*C. auris*) has emerged as a multidrug-resistant human fungal pathogen causing invasive nosocomial infections with high morbidity and mortality [1,2]. Its clinical isolates, which have been categorized into four major distinct geographical clades (referred to as Clade I~IV) [3], exhibit clade- and sub-clade specific properties associated with both virulence and drug resistance [4,5]. Except for the generally susceptible Clade II isolates, all remaining *C. auris* clinical isolates were reported to exhibit a diversified resistance to multiple drug classes including azoles and polyenes [6,7]. The high virulence and marked dissimilarity in response to antifungal drugs of the four phylogenetically distinct clades [8,9,10] has prompted the search for real-time/on-site unequivocal identification of *C. auris* at both clade and subclade levels. 

The root of the developed resistance of *C. auris* to the existing antifungal remedies resides in the lack of a real-time, sensitive, and reliable diagnostic tool to unequivocally identify it at the species, clade, and sub-clade levels. This has been the main cause of nosocomial outbreaks, which has made this pathogen a superbug. Only several years after its first isolation in 2009 [11], the whole-genome sequence of multiple *C. auris* clinical isolates became available, and the simultaneous emergence of different clades from various geographical regions could be revealed. *C. auris* is indeed hardly distinguishable from other, more common species of *Candida* by simply examining its phenotypic traits (e.g., morphology, colony appearance, pigmentation in chromogenic culture medium, etc.). Accordingly, the last decade has seen a rapid evolution of diagnostic methods for its identification. Precise *C. auris* speciation requires resorting to molecular methods, which include DNA sequencing [12], proteomics (i.e., matrix-assisted laser desorption/ionization time-of-flight mass spectrometry and MALDI-TOF) [13], and metabolomics (i.e., nuclear magnetic resonance and NMR) [14]. These methods are capable of unequivocally distinguishing *C. auris* from its close relatives, and further implementations are ongoing to distinguish among subclades and isolates from different geographical areas.

In principle, a full identification of *C. auris* at the clade/subclade level requires whole-genome DNA sequencing and multilocus sequence typing. These analyses greatly impact on costs and turnaround time for collection of the results. Accordingly, efforts have been made to refine genome-mapping criteria to save time and cut down costs. Advanced studies have recently succeeded in locating unique DNA sequence junctions that map clade-specific regions, a procedure greatly reducing the time necessary to locate the four major clades of *C. auris* [15]. Regarding proteomics, up-to-date MALDI-TOF databases are now available, which include *C. auris* strains from all four phylogenetic clades. Such advanced databases allow overcoming clade identification challenges with high identification scores [16]. Bruno et al. [17] have succeeded in the metabolomic NMR speciation of different *C. auris* clades through selectively collecting differences in the structure of their isolated mannans. Despite these improvements and newly obtained insights, the unique-DNA sequence-junction mapping still impacts labor costs, and MALDI-TOF proteomics and NMR metabolomics require elaborate sample preparation, sophisticate databases, and the availability of expensive equipment. For this body of reasons, a suitable procedure for on-site/real-time profiling of *C. auris* species/clades/subclades is nowadays unavailable to clinical practitioners.

As the number of *Candida* species with completely sequenced genomes increases, extensive research is being conducted in the search for the specific genes responsible for their virulence and drug resistance. Recent studies have revealed the presence of unique chemical fingerprints and metabolite profiles enabling not only *Candida* speciation but also the identification of functional links between metabolic and virulence traits of different species [17,18]. Owing to its close relationship to phenotype and its unique ability to probe complex biochemistry, analytical approaches to metabolomics have thus emerged as powerful tools for exploring the pathogenicity of *Candida* species. Confocal Raman spectroscopy (and related imaging) is a relatively new tool in metabolomics, which allows extracting and spatially visualizing biological information from living cultures of cells, yeasts, and bacteria with high spectral and spatial resolutions [19,20]. The main advantage of Raman spectroscopy over DNA-sequencing resides in its non-destructiveness and measurement swiftness, which position it as a unique chairside technology for sensitively profiling *Candida* species in nearly real time. Moreover, modern Raman instruments are relatively inexpensive as compared to MALDI-TOF or NMR devices and can be equipped with optical hardware that allows for the collection of maps of millions of spectra in minutes. Once empowered with deep learning approaches and multivariate statistical methods, the Raman spectra can be decomposed into a series of images representing chemical signatures of specific molecules [21]. Hyperspectral Raman images can thus locate and spatially quantify the presence of selected metabolites in time-lapse fashion without requiring the addition of exogenous markers, namely, with an agile procedure compatible with cell life.

In our previous Raman studies of *Candida* species/clades [22,23,24], we have interrogated spectral profiles in search of peculiar chemical fingerprints of speciation, virulence, and drug resistance. In addition to using machine-learning procedures, we have also proposed a new bioinformatics approach referred to as “Raman barcoding”. This approach converts a machine-learning-deconvoluted Raman spectrum into a barcode that facilitates electronic recordkeeping and translates molecular characteristics into information rapidly accessible by users. The present study builds upon those previous studies by showing that Raman spectroscopy is capable of accurately speciating all *C. auris* clades and subclades, while also locating links between their virulence, stress resistance, and metabolite structure. The presented Raman methodology opens a new path toward Raman metabolomics of *C. auris*, which not only enables the correct selection and patient customization of drug dosing, but also uniquely provides hints for developing new strategies in counteracting candidiasis/candidemia pathophysiology.

## 2. Experimental Procedures

### 2.1. Candida Auris Clades and Subclades

*C. auris* clades and their related subclades were provided by Teikyo University for a total of seven different cell samples, as follows: LSEM3690 (Clade I; South Asian), LSEM3682 and LSEM643T (Clade II; East Asian), LSEM3672 and LSEM3673 (Clade III; African), and LSEM3662 and LSEM3663 (Clade IV; South American). All clades were cultured in brain–heart infusion (BHI) broth (NISSUI PHARMACEUTICAL Co., Ltd., Tokyo, Japan) at 36 °C for 24 h under atmospheric pressure on a glass-bottom plate (MatTek Corporation, Ashland, MA, USA). Portions of the clade samples were then mounted in distilled water on a microscope slide (Matsunami Glass Ind., Ltd., Kishiwada, Osaka, Japan) and observed with a laser microscope (VK-x200 series, Keyence Co., Ltd., Osaka, Japan) at a magnification of 400~1000×. The remainder of the samples of each colony was characterized by Raman spectroscopy, and the spectra were analyzed and compared.

### 2.2. Raman Library, In Situ Raman Spectroscopy, and Raman Imaging

A library of reference Raman spectra was built by using 40 selected elementary compounds, including polysaccharides (e.g., chitin, β–1,3-glucans, β–1,6–glucans, α–1,3–glucans), mono– and disaccharides (e.g., trehalose, β–D–glucose, D–dextrose), lipids (e.g., triolein, trilinolein, 1,2–dipalmitoyl–L–α–lecithin), polyols (e.g., D–(+)–Arabitol and L–(−)–Arabitol), and other key molecules such as adenine, ergosterol, and glycine. The elementary compounds were selected and their spectra deconvoluted in order to feed a computer program designed to pick up a series of Gaussian–Lorentzian sub-bands from a library (cf. forthcoming Section 2.3). Key molecules were selected according to the previously published literature on the structure of *C. albicans* and other yeasts [25,26,27].

The reference α–1,3–glucan samples were kindly provided by Tokyo University [28]. All reference spectra stored in the library were collected with a high spectrally resolved Raman device (T-64000; Jobin-Ivon/Horiba Group, Kyoto, Japan) equipped with a nitrogen-cooled charge-coupled device detector (CCD-3500V, Jobin-Ivon/Horiba Group, Kyoto, Japan). The excitation source was the 514 nm line of an Ar-ion laser operating with a nominal power of 200 mW. The spectral resolution was better than 1 cm^−1^.

Raman spectra were collected in situ using a spectrometer specially designed for measurements on biological samples (LabRAM HR800, Horiba/Jobin-Yvon, Kyoto, Japan). A 20× objective lens was used throughout the experiments, and the optical circuit was set in confocal mode. High efficiency and high spectral resolution could be concurrently achieved by employing a holographic notch filter. The incoming light was a 532 nm solid-state laser source operating at 10 mW. A spectral resolution of better than 1 cm^−1^ was achieved by using an internal reference (neon emission) to calibrate the spectrometer at each measurement. The Raman-scattered light was monitored by means of a single monochromator interfaced with an air-cooled charge-coupled device (CCD) detector (Andor DV420-OE322; 1024 × 256 pixels). The acquisition time for a single spectrum was typically 10 s. Twenty spectra were collected at different locations over a total area of ~2 mm^2^ for each *Candida* sample and averaged in order to obtain a representative spectrum for each clade/subtype. 

Raman images were collected using a dedicated Raman device (RAMANtouch, Nanophoton Co., Minoo, Osaka, Japan) operated in microscopic measurement mode (100× lens) with confocal imaging capability in two dimensions. As a peculiarity, this Raman microscope is capable of achieving ultra-fast simultaneous image acquisition of up to 400 spectra in order to be compatible with cells’ lives. The excitation source was a 532 nm solid-state laser, and the spectral resolution was ~2 cm^−1^ (spectral pixel resolution equal to 0.3 cm^−1^/pixel) with an accuracy in a laser spot spatial location of 100 nm. Raman hyperspectral images were generated using commercially available software (Raman Viewer, Nanophoton Co., Minoo, Osaka, Japan). Raman images were generated using intensity ratios from normalized spectra. In order to minimize errors related to spectral resolution and possible shifts in band position, we used the average intensity of the pixels at the band nominal location ± 3 pixels, rather than single pixel intensity. Selected areas of the samples were scanned with a lateral displacement step of 500 nm for the laser focal point.

### 2.3. Machine Learning Algorithm for Spectral Deconvolution

Experimental spectra were preliminarily treated with a polynomial baseline subtraction and then deconvoluted into a series of Gaussian–Lorentzian sub-bands. The baseline subtraction procedure was performed using options available in commercial software (LabSpec 4.02, Horiba/Jobin-Yvon, Kyoto, Japan) by following the same criteria for all spectra collected on different clades. The automatic software applied a polynomial-fitting criterion for baseline subtraction. All spectra were analyzed for their relative intensity after normalization to the glucose ring signal (detected at ~478 cm^−1^). Detailed descriptions of spectral deconvolution criteria have been given in previous works [20,22]. Briefly, average spectra were fitted by means of an automatic solver, which exploited a linear polynomial expression of Gaussian–Lorentzian functions through a working algorithm iteratively run to match the experimental spectrum for minimum scatter (better than 95% confidence interval). A computer program was built up for selecting a series of deconvoluted sub-bands from pre-selected compounds belonging to the aforementioned library, including mono–, di–, and polysaccharides, specific sterol lipids, polyols, and other key molecules pre-selected according to the previously published literature on the structure of the *Candida* species. Following the pre-selection of elementary molecules from the library, the algorithm located the best fit to the experimental spectra by preserving relative intensities, spectral positions, and full-width at half-maximum values from each elementary compound (i.e., within ±3 cm^−1^; a boundary value selected by considering the resolution of the spectrometer and the possibility of slight alterations in molecular structure). Mathematical constraints on band positions, relative intensity, and bandwidths allow for a univocal deconvolution of the experimental spectra. When specific sub-bands appeared, which the solver could not properly fit according to the pre-selected library compounds, a new compound was selected from the library to match the unknown band and a new run of adjustments was performed for determining the intensity contributions of all elementary compounds.

Regarding the details of the machine learning algorithm and related training/validation procedures, we used a *K*-means clustering algorithm based on sets of 4 × 10^4^ spectra obtained from Raman imaging using arrays of 100 arrays of 400 spectra.

### 2.4. Chemometric Analysis and Barcoding

Statistical analyses and visualization of the large Raman data sets obtained during high-resolution imaging were performed according to principal component analysis (PCA). The number of spectra collected in Raman imaging was in the order of 10^6^ spectra per image. PCA analyses were carried out on the Origin software platform (OriginLab^®^ Co., Northampton, MA, USA) and enabled the display of statistical information with a set of “summary indices”, referred to as principal components PC1 and PC2.

A barcode was built from deconvoluted Raman spectra in order to enable efficient electronic recordkeeping and to increase data accessibility and structural characteristics of different species through apps and user-friendly software. Sequences of deconvoluted Gaussian–Lorentzian sub-bands were converted into barcodes by means of an algorithm assigning to each band a line with a thickness equal to 1/50 of the sub-band full width at half maximum and a distance from the successive line proportional to the sub-band area.

### 2.5. Statistical Analysis

The statistical relevance of the experimental data was analyzed by computing mean values and standard deviations. Their statistical validity was evaluated by applying the unpaired Student’s *t*-test [29]. For each sample, *p*-values < 0.05 and <0.01 were considered as statistically significant and labeled with one and two asterisks, respectively.

## 3. Experimental Results

### 3.1. Morphological Observations of the Studied Clades/Subclades

**Figure 1:**
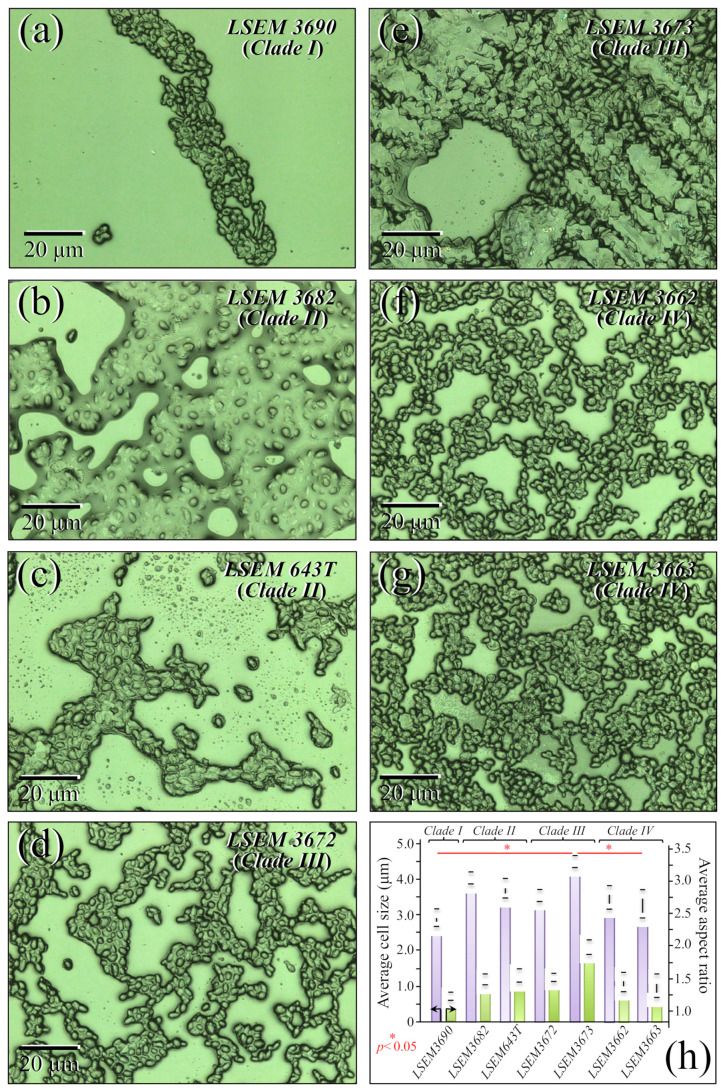
(**a**–**g**) Laser micrographs of the seven investigated clades/subclades of *C. auris* (four clades in total with related subclades; cf. labels in inset); (**h**) average cell size and aspect ratio as directly measured on the respective micrographs are shown with their respective standard deviations (statistically relevant differences according to statistical *t*-test validations with significance set at *p* < 0.05 and labeled with one asterisk; cf. labels in inset).

Figure 1a–g shows laser micrographs of four *C. auris* clades and related subclades (total of seven samples; cf. labels in inset). Cell size and aspect ratio, which were directly measured (and averaged) on the respective laser micrographs, showed some statistically relevant differences among the studied clades/subclades (Figure 1h; cf. standard deviations and related statistical *t*-test validations in inset). Cells of LSEM3673 (Clade III) were the largest in size (4.5 ± 0.2 μm in average) and the most elongated ones (aspect ratio equal to 2.0 ± 0.12), whereas those of LSEM3690 (Clade I) were the smallest in size (2.9 ± 0.25 μm) and almost roundish in shape (aspect ratio equal to 1.25 ± 0.06). Cells belonging to all remaining clades/subclades investigated presented intermediate average sizes within narrow intervals (3.3~4.0 μm and 1.3~1.6 for size and aspect ratio, respectively). The above morphological observations are in line with microscopy observations presented by other authors on *C. auris* clades [30,31,32]. However, so far, no direct comparisons for complete sets of *C. auris* clades/subclades cultured under exactly the same conditions could be found in the published literature. Despite the aforementioned morphological differences, it appears extremely difficult to speciate *C. auris* clades by means of mere morphological analyses. An important detail was that some specific subclades, such as LSEM3682 (Clade II) and LSEM3673 (Clade III), appeared to be abundantly embedded into an extracellular matrix (ECM), whereas others, i.e., both Clade IV subclades, preferred to aggregate into small clusters of cells without sharing any ECM. The observed ECM corresponded to the general description given by Flemming and Wingender [33] for *Candida* species: a polymeric gel-like hydrated three-dimensional structure in which the cells become partially immobilized. The ECM plays a number of different roles in the overall physiological behavior of *Candida* yeast cells. These include providing a defense against phagocytosis and obstructing drug diffusion. The ECM structure is both species- and environment-dependent, but, as a general characteristic, its major components are polysaccharides (glucans and mannans), monosaccharides (including) hexosamines, with additional fractions of proteins (and their glycosylated counterparts), phosphorus, and uronic acid [34,35]. The presence and roles of ECM will be discussed in more detail in the context of Raman analyses.

### 3.2. Raman Markers for Different Metabolites in C. auris Clades/Subclades

**Figure 2:**
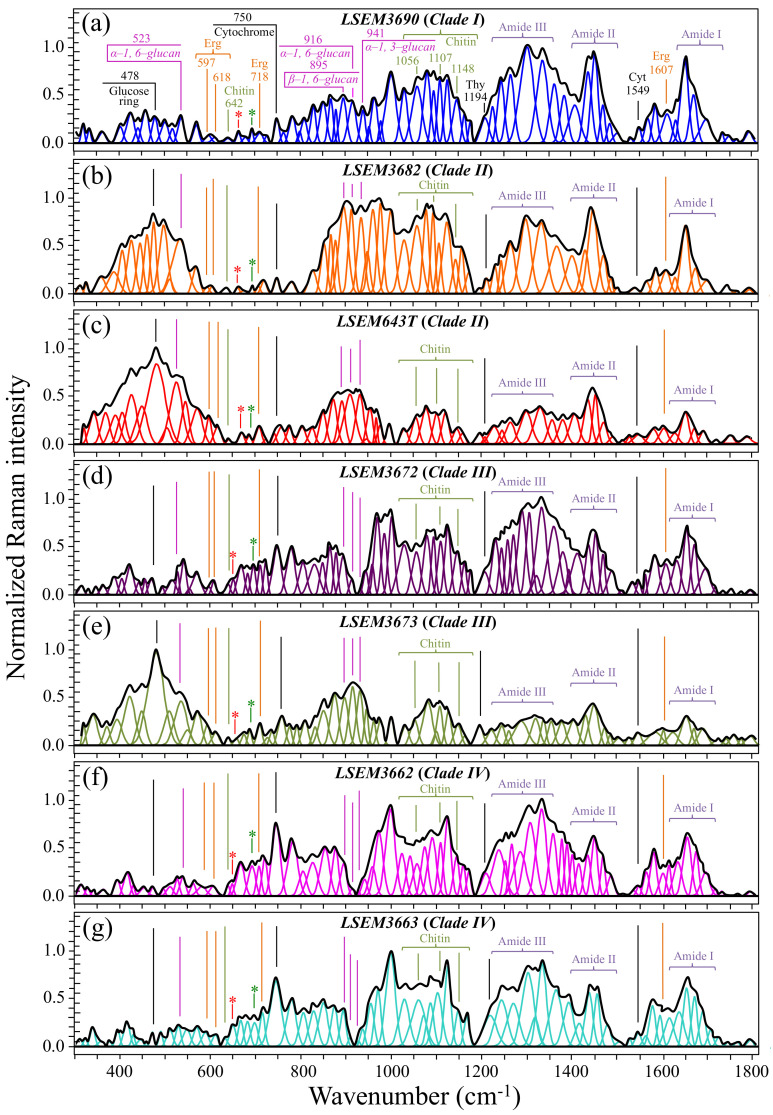
(**a**–**g**) Deconvoluted Raman spectra for the seven investigated samples of *C. auris* (cf. labels in inset). Each spectrum represents the average of 20 spectra collected with a 20× objective lens on each sample at different locations. Band assignments in inset are discussed in text (wavenumbers are in cm^−1^ units); red and green asterisks locate signals from ergosteroids and β-chitin allomorph, respectively.

Averaged and deconvoluted Raman spectra for the seven investigated clades/subclades of *C. auris* are shown in Figure 2a–g (cf. labels in inset to the respective spectra). Despite some morphological similarity, several bold differences among spectra could be spotted at a glance. The presence of morphological differences among the collected spectra suggests that Raman biochemical profiling could be possible for *C. auris* clades upon performing detailed spectroscopic analyses. A description of the main spectral differences is offered in the following.

*Biofilm-related signals*—One striking difference that immediately appeared in comparing spectra from different clades over the whole recorded wavenumber interval (Figure 2) was a marked heterogeneity in signal intensity in the low-frequency region between 350 and 600 cm^−1^. This region is mainly contributed to by signals related to glucose ring vibrations (C–O–C bending and glucose ring deformation modes) [34]. Remarkably, spectra with the strongest signal intensities in the 350~600 cm^−1^ region exactly corresponded to samples for which the presence of ECM could be observed (i.e., compare Figure 1b,c,e to Figure 2b,c,e, referring to LSEM3682 (Clade II), LSEM643T (Clade II), and LSEM3673 (Clade III), respectively). As described above, the ECM indeed consists of poly- and monosaccharides made of chained glucose rings incorporating and linked by C–O–C bonds [35,36]. However, though the glucose ring band at 478 cm^−1^ (C–O–C bending) was the most intense band in the recorded spectra of two of the three ECM-embedded subclades (i.e., LSEM643T (Clade II) and LSEM3673 (Clade III)), the spectrum of the third subclade (LSEM3682 (Clade II)) presented its strongest signal at around 895 cm^−1^, namely, in a spectral zone dominated by signals from β-glucans. This difference points at peculiar chemical fingerprints for the ECM of different clades and will be discussed later in the context of glucans’ signals.

**Figure 3:**
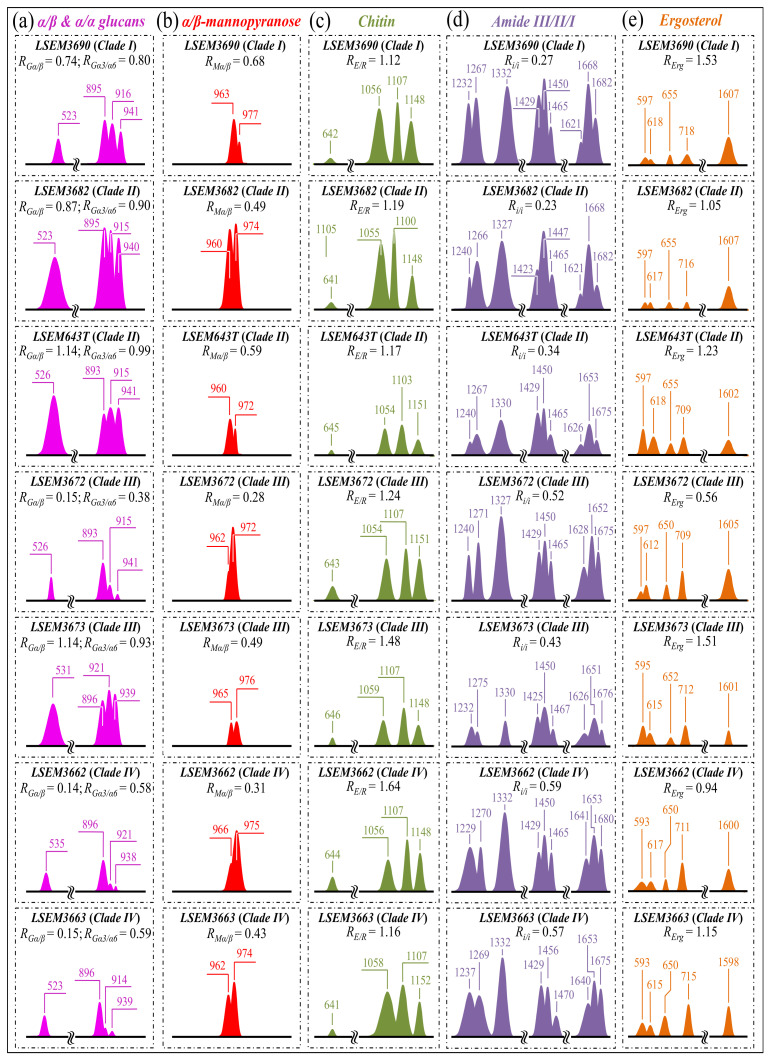
Deconvoluted band components (redrawn from the spectra in Figure 2) for: (**a**) α/β and α/α glucans; (**b**) α/β-mannopyranose; (**c**) chitin; (**d**) amide III/II/I; and (**e**) ergosterol/ergostane. In inset, the computed values are given for the selected Raman biochemical parameters: *R_Gα/β_* = *I*_932_/*I*_890_, *R_G_*_α3/α6_ = *I*_941_/*I*_919_, *R_Mα/β_* = *I*_960_/*I*_974_ + *I*_960_, *R_E/R_* = *I*_1107_/*I*_1054_, *R_i/i_* = *I*_1644_/*I*_1660_, and *R_Erg_* = *I*_597_/*I*_620_, respectively. The wavenumbers given in inset are in cm^−1^ units.

*Markers of cell energetic state*—An important marker of cell physiology can be identified in the band at ~750 cm^−1^ (cf. Figure 2). This Raman band, which arises from breathing vibrations of the pyrrole ring [37], is seen in all *C. auris* clades/subclades. However, it showed significantly different relative intensities depending on clades/subclades. The pyrrole ring is present in a number of different biomolecules, including tryptophan, cytochrome, chlorophyll, and lactic acid. In the case of Raman spectrum of *Candida* yeasts, the 750 cm^−1^ band has mainly been assigned to the pyrrole rings present in cytochrome *c* [38]. Cytochrome *c* is a widely investigated Raman-sensitive biomarker in cell physiology [39,40]. Variations in relative intensity of the pyrrole ring band at 750 cm^−1^ can be taken as representative of the fraction of cytochrome *c* present in the mitochondria of the *Candida* cells. This signal reflects the participation of haem Fe^2+^ molecules in non-apoptotic functions (e.g., adenosine triphosphate synthesis), and can be taken as an intrinsic measure of the energetic state of the *Candida* cells. The two subclades of Clade IV, which presented the strongest relative intensities for the 750 cm^−1^ signal (cf. Figure 2f,g), should thus be considered as the most energetically activated cells under the selected culture conditions. Interestingly, in all three subclades showing a large amount of ECM, the signal from the cytochrome *c* marker was quite weak, suggesting that cells embedded in ECM are not only immobilized but also energetically “dormant”. However, the Raman signal of cytochrome *c*, despite its importance as a marker of cell energetics, is not a suitable parameter for speciating *Candida* cells. This is due to its strong dependence on momentary cell metabolism and extrinsic stress states. Attempts are hereafter shown to locate a series of Raman spectroscopic parameters, which not only relate to intrinsic structural features, but are also independent (although not completely) of the momentary metabolic/stress state of *Candida* cells. Figure 3 highlights the deconvoluted band components, which directly relate to cell metabolites and serve to establish the set of Raman spectroscopic parameters selected in this study for evaluating the structural characteristics of different *C. auris* clades/subclades.

*Glucans*—The Raman spectra of glucans present clear fingerprints that allow for their recognition from their specific secondary structures [34]. Spectral marks for β–1,3–glucans and α–1,3–glucans can be found at ~890 cm^−1^ and ~941 cm^−1^, respectively. These bands have a common origin (C–O–C glycosidic linkage vibrations) but differ by ~50 cm^−1^ in wavenumber as a consequence of their different bond environment [41,42]. The intensity ratio of 941 to ~890 cm^−1^ signals, *R_G__α/β_* = *I*_941_/*I*_890_, is thus linked to the volumetric fraction of α– to β–glucans. The value of the ratio, *R_G__α/β_*, differed among *C. auris* clades/subclades (Figure 3a). The highest fraction of α–1,3–glucans (*R_G__α/β_* = 1.14) was found in LSEM643T (Clade II) and LSEM3673 (Clade III), and the lowest one was consistently found in both Clade IV subclades (*R_G__α/β_* = 0.14~0.15). A striking finding was that the other LSEM3672 subclade of Clade III showed a quite low value (*R_G__α/β_* = 0.15), proving that *C. auris* metabolomics is not only clade- but also strongly subclade-dependent. On the other hand, the *R_G__α/β_* values measured in LSEM3690 (Clade) I and LSEM3682 (Clade II) were both moderately high (*R_G__α/β_* = 0.74 and 0.87, respectively; cf. Figure 3a). A comparison of the three subclades embedded in ECM (i.e., LSEM3682 (Clade II), LSEM643T (Clade II), and LSEM6373 (Clade III)) reveals that their ECM glucan structures share the common characteristic of a relatively high fraction of α–glucans.

An additional spectroscopic parameter related to the glucan structure can be located in the ratio between Raman signals from α–1,3–glucans and α–1,6–glucans. The aforementioned Raman signal peculiar to the α–1,3–glucan structure (i.e., stretching of the C1–O–C3 inter-ring bonds located at 941 cm^−1^; cf. Figure 1) can be compared with the marker for the α–1,6–glucan structure found at 919 cm^−1^ (C1–O–C6 inter-ring stretching; cf. Figure 1). Accordingly, the Raman intensity ratio, *R_Gα3/α6_* = *I*_941_/*I*_919_, can be taken as a marker of the balance between different α–glucan structures. The *R_Gα3/α6_* ratio is important because it links to the solubility in water of the yeast cell walls: the higher the ratio, the less soluble the glucan layer [43,44,45]. The ability of a given *Candida* species to synthesize higher fractions of water-insoluble glucans (i.e., α–1,3–glucans) is crucial in the structural and dynamical performance of both EMC and cell walls. Figure 3a shows sub-band components and related *R_Gα3/α6_* ratios for the seven *C. auris* samples under investigation. The highest *R_Gα3/α6_* ratios were found in the subclades with the highest *R_Gα/β_* values, namely, LSEM643T (Clade II), LSEM3673 (Clade III), and LSEM3682 (Clade II) (0.99, 0.93, and 0.90, respectively). These are the three samples embedded in abundant ECM (cf. Figure 1). On the other hand, the two subclades of Clade IV, which displayed the lowest *R_Gα/β_* ratios, also shared the lowest *R_Gα3/α6_* values (i.e., 0.58 and 0.59). The mismatch between the two Clade III subclades, already found for the *R_Gα/β_* ratio, is thus also confirmed for the *R_Gα3/α6_* ratio, with values of 0.38 and 0.93 detected in LSEM3672 and LSEM3673, respectively. This confirms that the high *R_Gα3/α6_* value of the latter is related to the presence of ECM (similar to the two subclades of Clade II). In addition to its high level of insolubility in water, α–1,3–glucan is also a very rigid molecule. Therefore, LSEM 3682 (Clade II), LSEM643T (Clade II) and LSEM3673 (Clade III) are capable of building a very rigid and water-insoluble ECM structure, whereas the two Clade IV subclades lack ECM and share a relatively flexible glucan structure in their cell walls. This point will be examined in more detail in the forthcoming Section 4.

*Mannopyranose*—The spectral region between 950 and 1200 cm^−1^, which is characteristic of deformation modes of C–OH, C–CH, and O–CH groups, exhibits bands representative of ring conformation and relative orientation of their substituents [46]. The vibrational response in this wavenumber interval allows for distinguishing between the β and α anomers of D–mannose. More specifically, the intensity ratio, *R_Mα/__β_* = *I*_960_/*I*_974_ + *I*_960_, of the bands at ~960 and ~974 cm^−1^ corresponds to the anomeric proportions of α and β forms [47]. Figure 3b shows the band components of α– and β–mannopyranose for different *C. auris* clades/subclades as extracted from the respective spectra in Figure 2. The computed *R_Mα/__β_* ratios for each sample are given in the inset. Consistent with the results of glucans, the three subclades embedded in the ECM matrix (i.e., LSEM 3682 (Clade II), LSEM643T (Clade II) and LSEM3673 (Clade III)) displayed high *R_Mα/__β_* ratios (0.49, 0.59, and 0.49, respectively). However, the highest in fraction of α anomer of D–mannose was the LSEM3690 (Clade I) (*R_Mα/__β_* = 0.68), which lacks ECM (cf. Figure 1a). The lowest *R_Mα/__β_* ratio was recorded in LSEM3672 (Clade III) (0.28). The remaining samples showed intermediate values, again showing clear heterogeneity between subclades of the same clade (cf. values for Clade IV in Figure 3b), as in the case of glucans. In the cell wall structure, mannans are dislocated in the outer layer, and their different structures play different roles in enhancing virulence and reducing immunogenic exposure [48]. Accordingly, the *R_Mα/__β_* ratio represents an important parameter in *C. auris* cell metabolomics, allowing virulence and immunogenic behaviors to be rationalized and monitored toward effective therapies.

*Chitin backbone*—The spectral region between 1000 and 1200 cm^−1^ is representative of C–C–C and C–O–C stretching vibrations in polysaccharides [34]. The main peculiarity of the chitin structure, as compared to other polysaccharides, is the appearance of C–O–C bonds between neighboring rings [49]. This structural peculiarity confers on chitin distinctive vibrational characteristics, namely, the triplet at 1054, 1107, and 1147 cm^−1^, as well as the low-frequency band at 645 cm^−1^, which stand conspicuously free from overlap with other signals. Conversely, the band at ~1123 cm^−1^ represents a cumulative signal from C–C (and C–O) skeletal stretching in all polysaccharides. Regarding the aforementioned chitin-related triplet, it enables a distinction between C–O–C bonds located *within* and *between* rings. The stretching of C–O–C bonds *within rings* produces the Raman signal at 1054 cm^−1^, whereas C–O–C stretching *between rings* is seen at 1107 cm^−1^. Accordingly, the band intensity ratio, *R_E/R_* = *I*_1107_/*I*_1054_, can be defined as the “esterification ratio” and taken as representative of the ether-to-ring C–O–C bond fraction in chitin. According to this definition, the *R_E/R_* ratio reflects whether the chitin structure incorporates long crystalline chains. Chitin structures with higher *R_E/R_* ratios contain a higher fraction of ether C–O–C bonds, and thus they consist of longer chains and are expected to bestow higher crystallinity (as demonstrated later). Figure 3c shows the C–O–C triplet representative of chitin for the seven clades/subclades of *C. auris* investigated in comparison with their respective low-frequency band at 645 cm^−1^. The computed *R_E/R_* values are given in the inset. LSEM3662 (Clade IV) appears to possess the chitin structure with the highest *R_E/R_* ratio (1.64), followed by LSEM3673 (Clade III) (1.48) and LSEM3672 (Clade III) (1.24). The remaining subclades investigated all experienced lower *R_E/R_* values, ranging between 1.12 and 1.19.

*Chitin sub-structures*—Another peculiarity of the chitin structure, among other polysaccharides, is the presence of amide groups that partly replace OH groups as structural sub-units in the polymeric chain. Amides I, II, and III Raman signals, which appear in the wavenumber intervals 1600~1700 cm^−1^, 1400~1500 cm^−1^, and at around 1300 cm^−1^, respectively, are peculiar to the N-acetyl group of chitin (Figure 3d). Accordingly, the relative intensities of these signals are linked to the level of acetylation/deacetylation of the chitin structure in the membrane of different *Candida* species. The Amide I doublet at ~1644 and 1660 cm^−1^ represents two different types of hydrogen bonds in α-chitin crystals, namely, intermolecular bonds between two N-acetyl groups belonging to neighboring molecules (C=O^…^HN) and intramolecular bonds between N-acetyl and hydroxyl groups or between two N-acetyl groups (C=O^…^HO(C6) or C=O^…^HN) belonging to the same molecule, respectively [50,51,52]. A spectroscopic evaluation of this doublet allows for classifying the *C. auris* clades/subclades according to the degree of acetylation of their chitin structure. The relative intensity ratio, *R_i/i_* = *I*_1644_/*I*_1660_, namely, the Raman ratio of intermolecular-to-intramolecular hydrogen bonds, could be taken as a parameter measuring the degree of acetylation of α-chitin: the higher the ratio, the more acetylated the structure. As discussed later, a highly acetylated structure of chitin serves to reduce membrane permeability against external molecules, including drugs [23].

Additional signals might also be found in the Amide I region as shoulder bands at 1675~1685 and >1688 cm^−1^. These signals can be assigned to C=O stretching and associated with the presence of different chitin secondary structures or defects in small crystalline chitin fibrils, respectively [53]. The two Clade IV subclades showed the highest *R_i/i_* ratios (0.59 and 0.57 for LSEM3662 and LSEM3663, respectively), and LSEM3682 (Clade II) and LSEM3690 (Clade I) showed the lowest ones (0.23 and 0.27, respectively). The degree of acetylation, namely, the fraction of acetyl groups present in the α-chitin structure, is linked to the crystalline structure of chitin, because a high degree of acetylation stabilizes the highly stable α-chitin crystalline structure through a network of hydrogen bonds that the amine acetyl groups form with NH groups [53,54,55]. Conversely, chitins with low degrees of acetylation and, thus, with a higher probability of primary amine groups in their structure are more likely to take different allomorphs or more defective structures (with high permeability). 

As mentioned above, the structure of α-chitin is stabilized by the presence of both intra- and inter-chain hydrogen bonds [50,51,52,56,57,58,59]; however, the former type of hydrogen bond could also be present in all other chitin allomorphs. According to Kaya et al. [58], the Raman spectra of α–, β–, and γ–chitin pure allomorphs present very few spectral details that could allow allomorphic differentiation. The machine learning procedure adopted in this paper located only one relatively hidden but still resolvable band at ~694 cm^−1^ that could unequivocally represent the β–chitin allomorph (located with a green asterisk in Figure 2). The relative intensity of this band, which arises from ring deformation [49], was the strongest in the spectrum of LSEM3672 (Clade III) and both samples of Clade IV, while being similarly weak in all other investigated samples.

*Ergosterol*—The two low-frequency bands located at ~597 and 620 cm^−1^ are related to in-plane ring deformation modes and are mainly (>95%) contributed by membrane sterols [60]. Fingerprint Raman signals related to the ergosterol structure are the two C=C stretching bands at 1602 cm^−1^ (within rings) and 1666 cm^−1^ (in acyl chains). However, only the former band can be clearly detected (as a shoulder) in the *C. auris* spectra, whereas the latter strongly overlaps with Amide I signals and is not univocally resolvable. Further, the relatively intense and isolate band at ~713 cm^−1^ is contributed by ergosterol (cumulative of C–H bending and ring deformation) [60], but it also contains significant contributions from D–arabitol, chitin, and adenine signals. Figure 3e shows the sterol-related sub-bands detected in the seven investigated *C. auris* clades/subclades. An important finding was that the doublet at 597/620 cm^−1^ showed a wide range of variability with respect to its relative intensity among the investigated samples. In pure ergosterol, the low-frequency band is more intense than the high-frequency one, and vice versa in ergostane [61]. Concurrently, an additional band at ~650 cm^−1^ appeared in the spectra (cf. red asterisk in Figure 2), which represents an additional ring vibration only ascribable to ergosteroids. Moreover, a tendency to lower intensity was noticed for the 1604 cm^−1^ stretching mode of –C=C– bonds in the second sterol ring when the 597 cm^−1^ band displayed intensity higher than the 620 cm^−1^ one. These three spectroscopic characteristics suggest the presence of a fraction of ergostane-type steroid structures (besides ergosterol) in all studied clades/subclades (cf. Figure 3e). According to the above description, one possible parameter for classifying ergosterol and its derivatives in different *C. auris* samples is the ratio between the relative intensities of the 597/620 cm^−1^ doublet, *R_Erg_* = *I*_597_/*I*_620_. *R_Erg_* values >1 are characteristic of a membrane structure with a preponderant ergosterol fraction, as in the case of LSEM3690 (Clade I), LSEM3673 (Clade III), LSEM643T (Clade II), and LSEM3682 (Clade II) in decreasing order (cf. *R_Erg_* values in inset to Figure 3e). Conversely, *R_Erg_* values <1 should mark an increasing predominance of the ergostane-type steroid structures, as in the case of LSEM3672 (Clade III).

**Figure 4:**
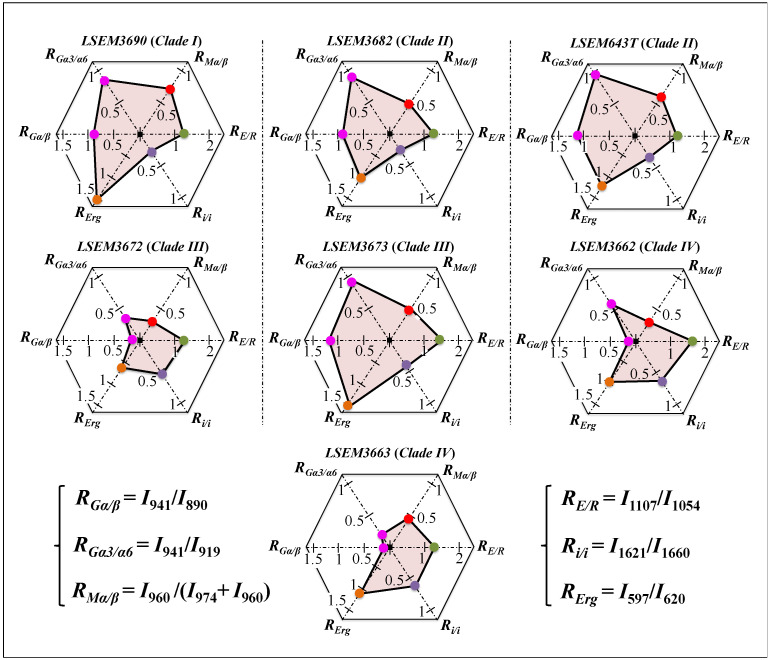
Summary of the computed Raman biochemical parameters *R_Gα/β_* = *I*_932_/*I*_890_, *R_G_*_α3/α6_ = *I*_941_/*I*_919_, *R_Mα/β_* = *I*_960_/*I*_974_ + *I*_960_, *R_E/R_* = *I*_1107_/*I*_1054_, *R_i/i_* = *I*_1644_/*I*_1660_, and *R_Erg_* = *I*_597_/*I*_620_, which are shown in a polar plot for each *C. auris* sample investigated (cf. labels in inset).

In summary, the above analysis of deconvoluted Raman spectra collected on *C. auris* clades/subclades revealed significant differences and located a series of spectroscopic parameters (referred to as Raman-biochemical parameters, henceforth) representing vibrational fingerprints of polysaccharides and lipids. The computed Raman biochemical parameters are summarized in Figure 4 in polar plots for the seven *C. auris* samples investigated. The plots vividly reflect a variety in the fractions of primary metabolites, which are linked to key structural characteristics of *C. auris* clades/subclades and reveal a breadth in their functional diversity.

### 3.3. Raman Imaging and Quantitative Calibrations of Metabolite Fractions 

In addition to spatially visualizing metabolites in *C. auris* cell cultures, Raman imaging performed with sub-micrometric spatial resolution made a large number of spectra (in the order of 10^6^) available, which served to typify the clades/subclades with respect to the five Raman biochemical parameters described in the previous section with high statistical validity. The Raman-imaging approach is complementary to the obtainment of representative Raman spectra for each subclade upon averaging over 20 spectra collected with a low-magnification optical probe (20×), including multiple cells (10~20) in each acquired spectrum (i.e., as those shown in Figure 2). A comparison between average values of Raman biochemical parameters computed by averaging spectra from Raman images and spectra collected with the low-magnification (20×) probe showed agreement within 10%, thus validating the data shown in Figure 4. Spectra from Raman imaging were matched with calibration plots (obtained from previous work) in order to link the five Raman biochemical parameters, *R_G_*_α3/α6_ = *I*_941_/*I*_919_, *R_Mα/__β_* = *I*_960_/*I*_974_ + *I*_960_, *R_E/R_* = *I*_1107_/*I*_1054_, *R_i/i_* = *I*_1644_/*I*_1660_, and *R_Erg_* = *I*_597_/*I*_620_, to the actual fraction of α–1,3–glucans, F_α3_; the α–D–mannopyranose fraction, F_M_; the degree of chitin crystallinity, D_C_; the degree of chitin acetylation, D_A_; and the fraction of ergostane-type steroids, F_Es_, respectively. 

**Figure 5:**
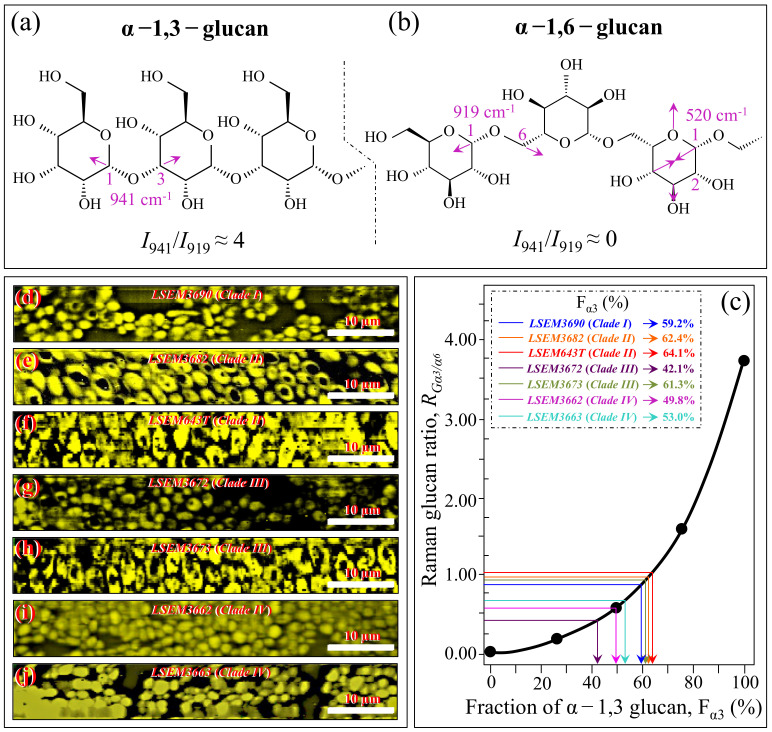
Molecular structure of (**a**) α–1,3–glucan and (**b**) α–1,6–glucan (with their respective Raman fingerprint vibrations); (**c**) Raman calibration plot of the *R_Gα3/α6_* = *I*_941_/*I*_919_ intensity ratio vs. fractions of α–1,3–glucan, F_α3_ (data from data in Ref. [24]; (**d**–**j**): Raman images of the *R_Gα3/α6_* ratio collected on different *C. auris* samples (cf. labels). The average *R_Gα3/α6_* value for each image locates the respective F_α3_ value on the calibration plot (**c**) (cf. values shown in inset given in %).

Figure 5a,b shows the molecular structures of α–1,3–glucan and α–1,6–glucan (with their respective Raman fingerprint vibrations), respectively. The results of a spectroscopic calibration performed on samples obtained by intentionally mixing α–1,6–glucan with increasing fractions of α–1,3–glucan and recording their *R_Gα3/α6_* = *I*_941_/*I*_919_ intensity ratio are shown in Figure 5c [24]. Figure 5d–j shows Raman images of the *R_Gα3/α6_* ratio collected on different *C. auris* samples (cf. labels). Upon entering the average *R_Gα3/α6_* value computed for each image in the plot of Figure 5c, one obtains the respective fractions of α–1,3–glucan, F_α3_ (cf. values shown in inset). The monotonic character of the *R_Gα3/_*_α6_–F_α3_ relationship preserves the order described in the previous section (i.e., labels in inset to Figure 3a), but its exponential character amplifies the differences in the *R_Gα3/α6_* ratio among different clades/subclades. The three samples embedded in abundant ECM (i.e., LSEM643T (Clade II), LSEM3673 (Clade III), and LSEM3682 (Clade II); cf. Figure 1) showed F_α3_ values >60%. However, LSEM3690 (Clade I) also showed a F_α3_ value as high as 59.2%, despite not showing ECM. On the other hand, the lowest amount of α–1,3–glucans, recorded in LSEM3672 (Clade III), was computed as 42.1%. The two Clade IV subclades displayed intermediate F_α3_ values at around 50%.

**Figure 6:**
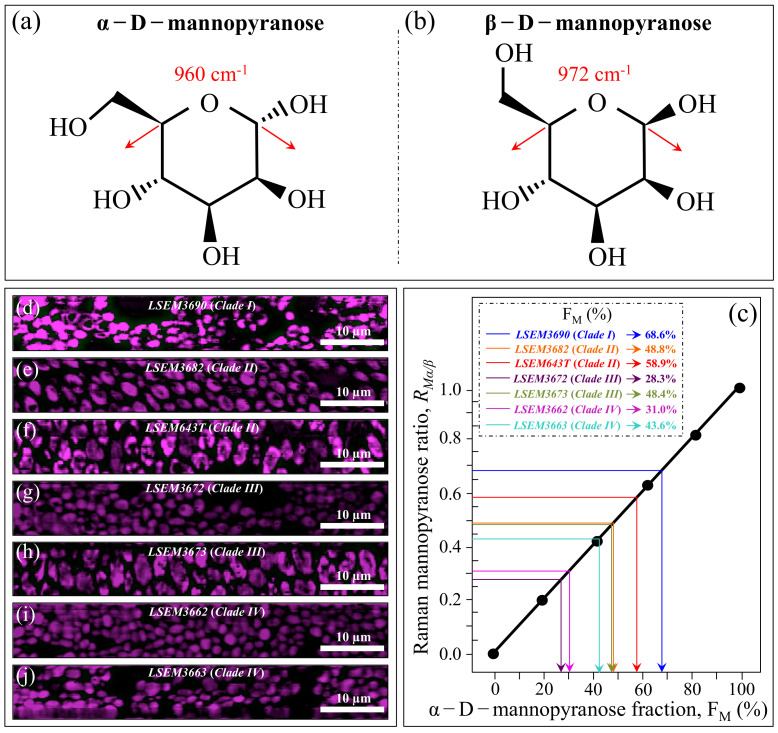
Molecular structure of (**a**) α–D–mannopyranose and (**b**) β–D–mannopyranose (with their respective Raman fingerprint vibrations); (**c**) Raman calibration plot of the *R_Mα/β_* = *I*_960_/*I*_974_ + *I*_960_ intensity ratio vs. fractions of α–D–mannopyranose fraction, F_M_ (data from data in Ref. [47]); (**d**–**j**): Raman images of the *R_Mα/β_* ratio collected on different *C. auris* samples (cf. labels). The average *R_Mα/β_* value for each image locates the respective F_M_ value on the calibration plot (**c**) (cf. values shown in inset given in %).

Figure 6a,b shows the structure of α–D–mannopyranose and β–D–mannopyranose (with their respective Raman fingerprint vibrations), respectively. In (c), a calibration plot is given, which links the Raman mannopyranose ratio, *R_Mα/β_* = *I*_960_/*I*_974_ + *I*_960_, to the α–D–mannopyranose fraction, F_M_. The linear plot is based on data reported in Ref. [48]. Figure 6d–j shows Raman images of the *R_Mα/β_* ratio recorded on the investigated *C. auris* clades/subclades (cf. labels). Average *R_Mα/β_* values were computed for each image and introduced in Figure 6c to obtain the respective α–D–mannopyranose fraction, F_M_ (cf. values shown in inset). The linear calibration plot unveils a trend similar to that shown for α–1,3–glucans, with the three ECM-embedded subclades (LSEM643T (Clade II), LSEM3682 (Clade II), and LSEM3673 (Clade III)) showing relatively high F_M_ fractions (i.e., 58.9, 48.8, and 48.4%, respectively). However, the highest fraction was recorded for the ECM-free LSEM3690 (Clade I) (68.6%), thus suggesting that α–D–mannopyranose molecules in this subclade predominantly belong to cell walls. Of note was also that, as compared with the fraction F_α3_ of α–1,3–glucans, the α–D–mannopyranose fraction, F_M_, fluctuated over a wider (i.e., nearly double) interval among different *C. auris* samples. The minimum F_M_ values were displayed for LSEM3672 (Clade III) (28.3%), thus confirming the possibility of wide fluctuations between samples belonging to the same clade as further proof of intra-clade functional diversity.

**Figure 7:**
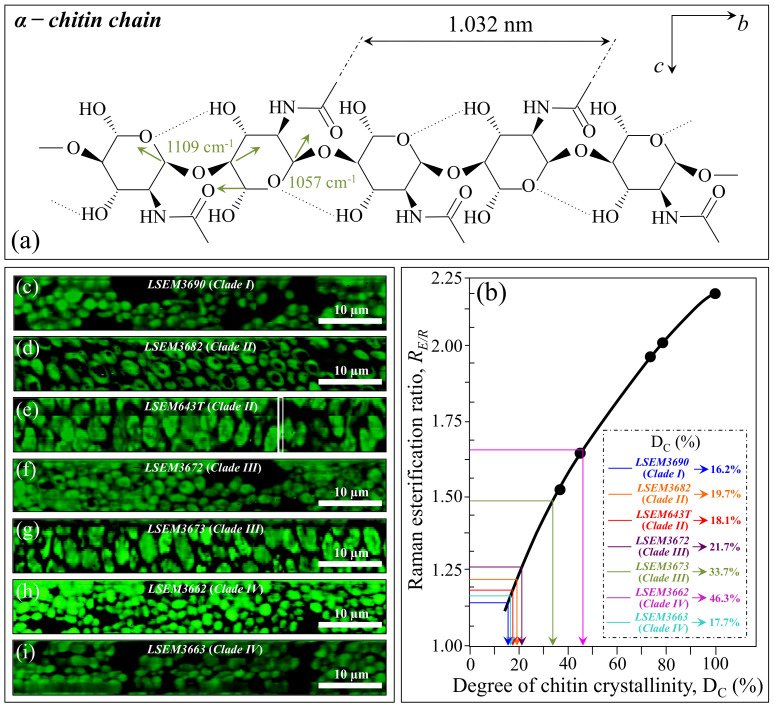
Molecular structure of (**a**) α–chitin (with the respective Raman fingerprint vibrations for inter- and intra-chain C–O–C bonds); (**c**) Raman calibration plot of the *R_E/R_* = *I*_1107_/*I*_1054_ intensity ratio vs. degree of chitin crystallinity, D_C_ (built from data in Ref. [59]); (**c**–**i**): Raman images of the *R_E/R_* ratio collected on different *C. auris* samples (cf. labels). The average *R_E/R_* value for each image locates the respective D_C_ value on the calibration plot (**b**) (cf. values shown in inset given in %).

In Figure 7a, the chain structure of α-chitin is shown together with its characteristic vibrational modes of inter- and intra-ring C–O–C bonds. Binias et al. [59] systematically compared Raman spectra of a series of chitin compounds with decreasing chain lengths with that of a fully crystalline α-chitin compound [62,63]. The computed *R_E/R_* = *I*_1107_/*I*_1054_ intensity ratio from the data in Ref. [59] (after normalization to the ether C–O–C stretching signal at 1109 cm^−1^) is plotted in (b) as a function of crystallinity values, D_C_ (determined by wide-angle X-ray diffraction measurements). As seen, the *R_E/R_* ratio sharply increased with increasing chitin crystallinity. Figure 7c–i show Raman images of the *R_E/R_* ratio collected on different *C. auris* samples (cf. labels). The average *R_E/R_* values computed for each image were then inserted into the plot of Figure 7b in order to retrieve the respective crystallinity fractions, D_C_ (cf. values shown in inset). Except for LSEM3662 (Clade IV) and LSEM3673 (Clade III) (i.e., the highest and second-highest in crystallinity with 46.3 and 33.7%, respectively), all other *C. auris* samples displayed relatively low crystallinity values (within the interval 16~22%). Since the length of a chitin dimer is 1.032 nm [52] and the average length of a standard α–chitin microfibril is ~33 nm, a chain of fully crystalline α-chitin contains in average 64 residues [48]. Assuming that the average chain length is proportional to the crystallinity value, *C. auris* samples with D_C_ ~20% or less likely only incorporate a small subgroup of crystalline α-chitin allomorph to form rigid microfibrils, whereas the remainders might take a different allomorphic structure or lie in a disordered state. This point will be detailed in Section 4.

**Figure 8:**
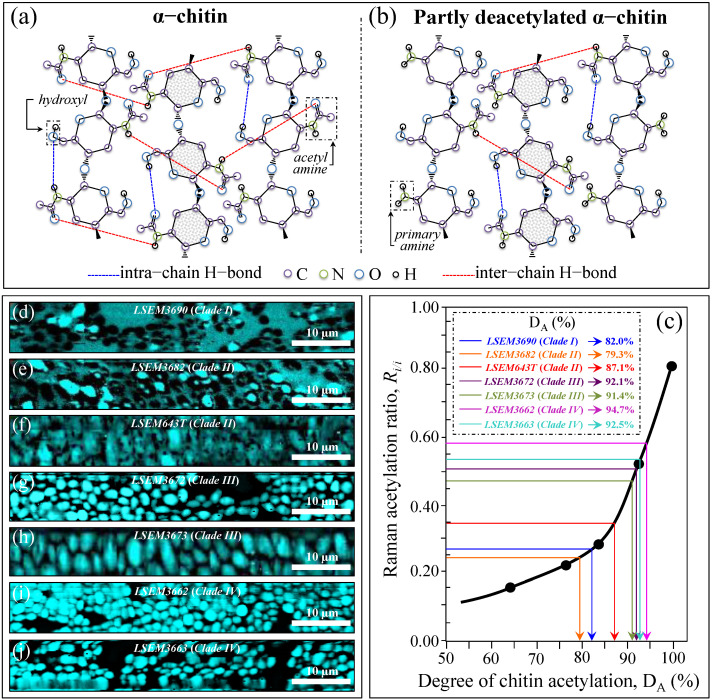
Molecular structure of (**a**) α–chitin and (**b**) partly deacetylated α–chitin (with their characteristic hydrogen bonds and molecular units); (**c**) Raman calibration plot of the *R_i/i_* = *I*_1644_/*I*_1660_ intensity ratio vs. degree of chitin acetylation, D_A_ (data from data in Ref. [51]); (**d**–**j**): Raman images of the *R_i/i_* ratio collected on different *C. auris* samples (cf. labels). The average *R_i/i_* value for each image locates the respective D_A_ value on the calibration plot (**c**) (cf. values shown in inset given in %).

Figure 8a,b shows the secondary structure of crystalline α-chitin (with its intermolecular (C=O^…^HN) and intramolecular (C=O^…^HO(C6) or C=O^…^HN hydrogen bonds) and the structure of a partly deacetylated α-chitin including primary amine groups to replace acetyl amines. Zhang et al. [51] proposed a Raman spectroscopic procedure to characterize α-chitin structures with respect to their degree of acetylation. The Amide I spectra of increasingly deacetylated chitin structures (i.e., containing decreasing fractions of acetyl groups) were used to link the structure-dependent vibrational characteristics to the degree of chitin acetylation, D_A_ [51]. A plot of the relative intensity ratio, *R_i/i_* = *I*_1644_/*I*_1660_, referred to as the Raman ratio of intermolecular-to-intramolecular hydrogen bonds as a function of D_A_ is given in Figure 8c, as obtained according to the data in Ref. [51]. The calibration plot, which shows an exponential character, allows for quantifying the degree of acetylation of the chitin structures of *C. auris* samples. Figure 8d–j shows maps of the *R_i/i_* ratio for different *C. auris* samples (cf. labels), from which average values were computed and matched with the calibration plot in Figure 8c. All clades/subclades experienced relatively high D_A_ values (i.e., comprised in the interval 79~95%), with the highest and lowest values being recorded for LSEM3662 (Clade IV) (94.7%) and LSEM3682 (Clade II) (79.3%), respectively. Similar to other metabolite-related parameters, D_A_ also showed differences between subclades of the same clade (cf. Clade II values in inset to Figure 8c).

**Figure 9:**
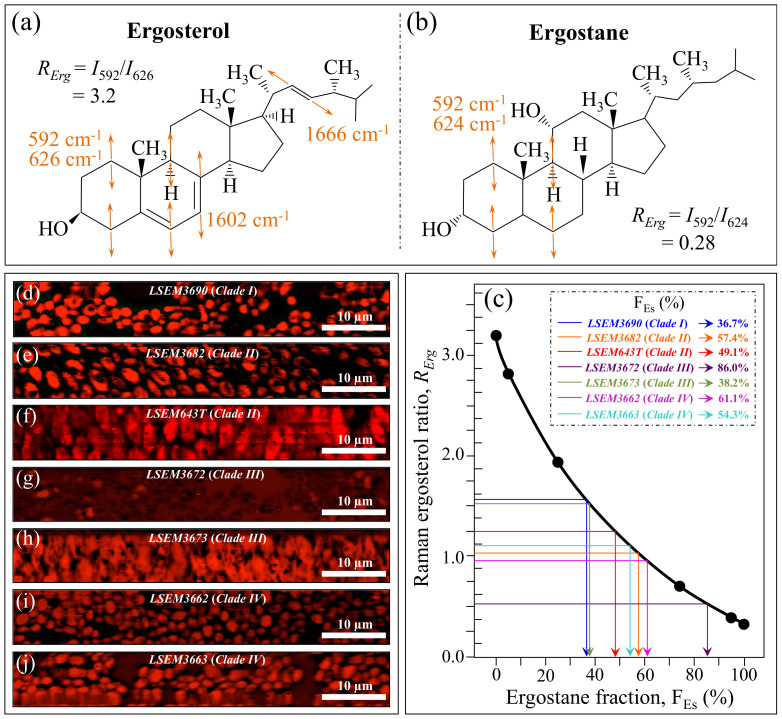
Molecular structure of (**a**) ergosterol and (**b**) ergostane (with the respective Raman fingerprint vibrations); (**c**) Raman calibration plot of the *R_Erg_* = *I*_597_/*I*_620_ intensity ratio vs. ergostane fraction, F_Es_ (built from data in Ref. [61]); (**d**–**j**): Raman images of the *R_Erg_* ratio collected on different *C. auris* samples (cf. labels). The average *R_Erg_* value for each image locates the respective F_Es_ value on the calibration plot (**c**) (cf. values shown in inset given in %).

Figure 9a,b shows drafts of the structure of ergosterol and ergostane structures (with their fingerprint Raman vibrational modes), respectively. Assuming the doublet at 597/620 cm^−1^ as representative of steroid sub-structures (as explained above), the *R_Erg_* = *I*_597_/*I*_620_ ratio was plotted as a function of the ergostane fraction, F_Es_, (Figure 9c) according to the data reported in Ref. [58]. In Figure 9d–j, maps of the *R_Erg_* ratio are given for different *C. auris* samples (cf. labels in inset). Average values were computed for each map and matched with the calibration plot in Figure 9c to obtain the respective F_Es_ values. In line with previous reports on the sterol composition of other *Candida* species [64,65,66,67,68], a wide variety of F_Es_ values could also be found in clades/subclades belonging to the *C. auris* species. LSEM3672 (Clade III) and LSEM3690 (Clade I) were the richest and poorest samples in ergostane (F_Es_ = 86.0% and 36.7%), respectively. These differences in sterol composition can greatly impact the drug resistance (especially amphotericin B) of different clades/subclades, as discussed in a later section.

**Figure 10:**
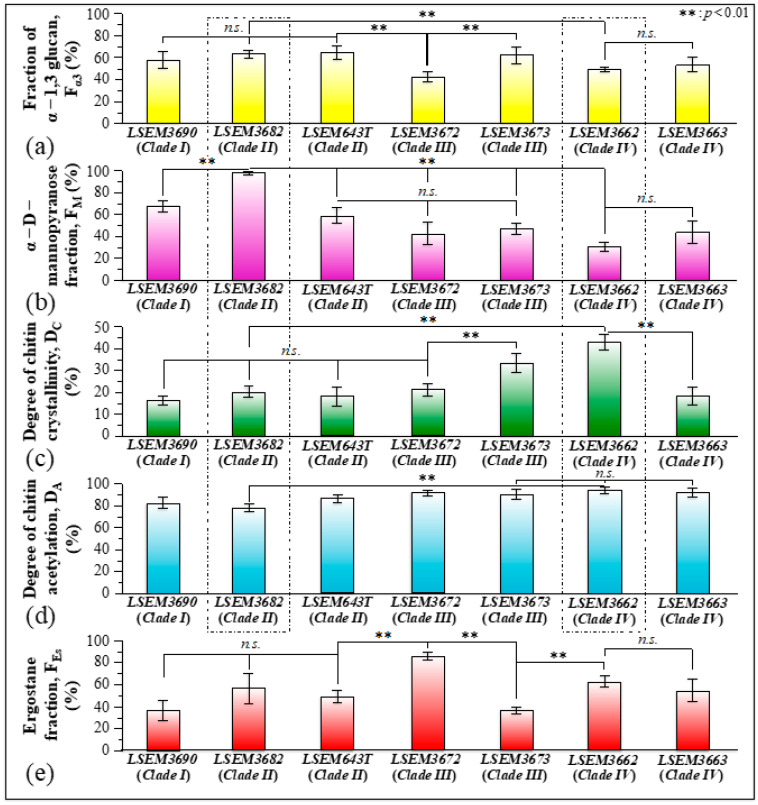
Metabolite fractional parameters (**a**–**e**): F_α3_, F_M_, D_C_, D_A_, and F_Es_ (cf. labels), as determined from Raman experiments on the investigated *C. auris* samples. Standard deviations are indicated in inset together with statistical evaluations performed on Raman maps collected at different locations (*n* = 3; with significance *p* < 0.01 labeled with **).

Figure 10a–e summarizes the metabolite fractional parameters F_α3_, F_M_, D_C_, D_A_, and F_Es_, respectively, as determined from Raman experiments on the *C. auris* samples investigated in this study. Standard deviations are indicated in the inset together with statistical evaluations performed on Raman maps collected at different locations (*n* = 3; statistical significance set at *p* < 0.01). The potential impact of such wide variations in cell metabolites on virulence and drug resistance will be discussed in the forthcoming Section 4.1 in comparison with the published literature.

### 3.4. Chemometric Results and Barcode Identification

**Figure 11:**
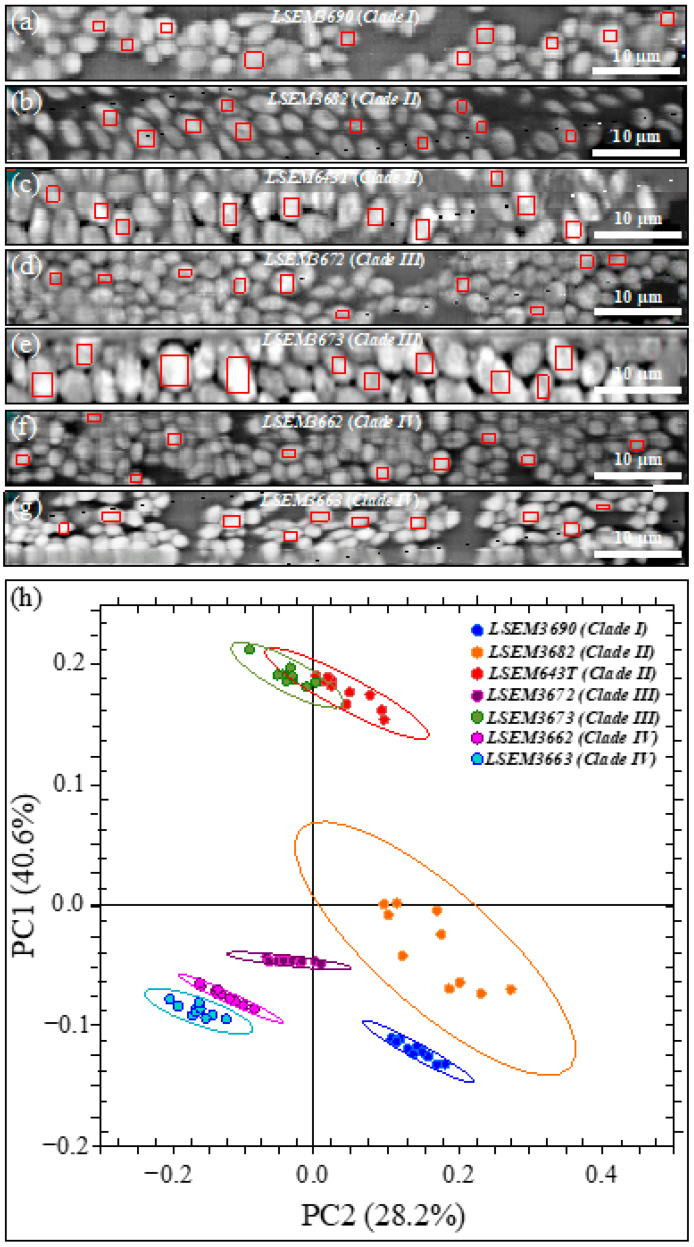
Images of the seven *C. auris* samples investigated (**a**–**g**) (cf. labels in inset). On each map, ten locations 10~20 μm^2^ in size are selected, in which 200~400 Raman spectra are collected and averaged to obtain a representative spectrum for each selected location; (**h**) PCA analyses of 10 average spectra in the Raman spectral region 300~1800 cm^−1^.

Images of the seven *C. auris* clades/subclades investigated are shown in Figure 11a–g (cf. labels in inset). On each map, ten locations 10~20 μm^2^ in size were selected (cf. red squares in each image), in which 200~400 Raman spectra were collected and averaged to obtain a representative spectrum for each selected location. The obtained 10 (average) spectra per each sample were then subjected to PCA analyses. Figure 11h shows the loading vectors of the first and second principal components (PC1 and PC2) as computed over the entire Raman spectral region 300~1800 cm^−1^. This plot shows that PCA analysis is capable of separating all *C. auris* clades/subclades except for the LSEM643T (Clade II) and LSEM3673 (Clade III). The present application of the PCA statistical method reduces the dimensionality of the Raman data matrix to two orthogonal variables and provides evidence for the usefulness and limitations of a standard chemometric analysis in identifying *C. auris* clades/subclades upon discriminating among their Raman spectra.

**Figure 12:**
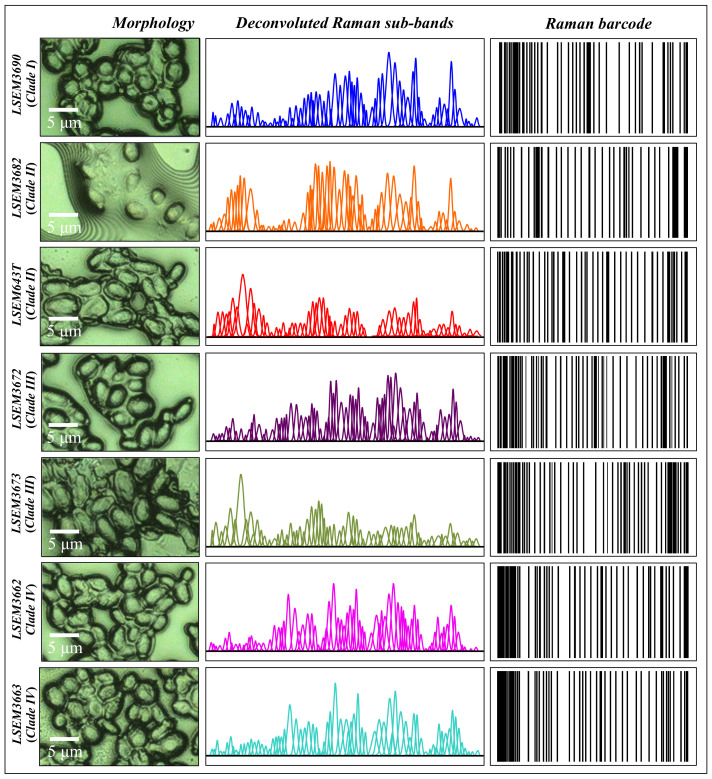
From left to right: high-resolution micrographs of different *C. auris* clades/subclades (cf. labels in inset), series of Gaussian–Lorentzian subclades (i.e., the same as those shown in the deconvoluted spectra of Figure 2) obtained from a machine-learning-based spectral deconvolution (cf. Section 2.3) for each sample, and Raman barcodes computed from the respective sub-band sequence according to the algorithm described in Section 2.4.

In order to overcome speciation deficiencies, Raman barcoding was proposed in previous studies as an alternative approach to chemometric PCA analyses [24,69,70]. In those previous studies, the Raman barcode approach allowed for obtaining a greater depth in capturing structural details than merely comparing the spectral morphology, as done in PCA analyses. Here, the barcode approach was applied to average spectra obtained from a series of 20 collected with high spectral resolution using a 20× optical lens on each *C. auris* sample. Figure 12 shows, side-by-side to high-resolution micrographs of subclade cells (cf. labels in inset), the respective series of Gaussian–Lorentzian sub-bands (i.e., the same as those shown in the deconvoluted spectra of Figure 2) obtained from machine-learning-based spectral deconvolution (as described in Section 2.3) for each sample under scrutiny. Raman barcodes (displayed on the right side of Figure 12) were then built up for each sub-band sequence according to the algorithm described in Section 2.4. As seen, the Raman barcodes, which contained subtle details of metabolite structures, amplified the differences between *C. auris* clades/subclades. Once translated into line patterns, such spectral differences became clearly distinguishable by the naked eye for all subclades, including LSEM643T (Clade II) and LSEM3673 (Clade III) (cf. barcodes in Figure 12), which were indistinguishable by PCA analysis.

## 4. Discussion

### 4.1. Cell Wall Structure and Stress-Resistance Strategies

As common to all *Candida* species [71,72], *C. auris* clades are also capable of building up ECM and structuring their cell walls in different ways in order to resist environmental conditions, to counteract drug effects, and to evade immune recognition. *Candida* species producing polymeric ECM generally exhibit lower mobility, slowed down energetics (cf. also Section 3.1), and higher resistance to antimicrobial treatments [73]. Different *Candida* species exhibit ECM formation strategies that vary with respect to both morphology and chemistry in order to potentiate the establishment of unyielding infections in the human host [74]. *Candida* cells are also quite efficient in constantly monitoring the integrity of the protective glucan/chitin structure of their cell walls, in order to minutely regulate the level of flexibility needed to permit turgor-driven and (osmotic) stress-driven cell expansion while preventing cell breakage [72]. Through minutely tailoring their compositional gradients and structural assembly, *Candida* cells maintain the delicate balance between rigidity and compliance of their walls. The Raman metabolomics data shown in Figure 10 indeed reveal different strategies between *C. auris* subclades producing ECM, as well as fundamental differences in the chemical structure of cell walls among different *C. auris* subclades. Raman analyses also suggest that both balance and redundancy exist in the strategies employed by different subclades to assemble their cell walls.

An instructive exercise in discussing different survivorship strategies and structural differences could reside in comparing LSEM3682 (Clade II) and LSEM3662 (Clade IV) with respect to both their propensity to produce ECM and composition of their cell walls. These two samples might be considered the most contrastive among *C. auris* subclades in terms of polysaccharides structural characteristics. With reference to the four quantitative parameters located as characteristic of cell wall structure (i.e., the fraction of α–1,3 glucan, F_α3_; the α–D–mannopyranose fraction, F_M_; the degree of chitin crystallinity, D_C_; and the degree of chitin acetylation, D_A_; cf. plots within broken lines in Figure 10), LSEM3662 (Clade IV) cells displayed the highest values in D_C_ and D_A_ among the investigated *C. auris* samples, whereas LSEM3682 (Clade II) showed the lowest values with respect to both parameters. The low degree of α-chitin crystallinity and the scarce presence of hydrogen bonds between its antiparallel chains confer high cell walls’ flexibility to the Clade II sample. Clearly, the LSEM3682 (Clade II) roots its surviving strategy in building copious ECM with a substantial fraction of stiff and insoluble α-glucan structure, which makes its F_α3_ value the second-highest among *C. auris* samples (i.e., after LSEM643T (Clade II) and LSEM3673 (Clade III), both also embedded in ECM; cf. Figure 10)). Note, however, that though LSEM643T (Clade II) shows an apparently weaker chitin structure (i.e., relatively low crystallinity, D_C_, and degree of acetylation, D_A_), LSEM 3673 (Clade III) possesses relatively high values of both chitin parameters, thus displaying strategic redundancy in concurrently producing ECM and strengthening the chitin structure of its cell walls.

ECM provides the yeast cells with a stiff shield, since the α–1,3–glucan polymorph is by far the most rigid molecule among polysaccharide structures (i.e., stiffer than chitin) [72]. Tailoring the ECM glucan structure is thus a quite effective ploy used by *Candida* cells in survivorship. Moreover, the α–1,3–glycosidic linkages are water-insoluble, as compared with water-soluble α–1,6–glycosidic linkages [43,44,45]. Note also that chitin and α–1,3–glucan intertwine to form a rigid and hydrophobic scaffold surrounded by a matrix of pliable and hydrated matrix of β–glucans [72]. Therefore, the Raman analysis revealed that LSEM3682 (Clade II) and LSEM3662 (Clade IV) cells have developed completely different strategies to balance stiffness, hydrophobicity, and impermeability in their cell walls. Such different strategies in turn lead to differences in resistance toward specific environmental stresses, which include cationic/osmotic, oxidative, and nitrosative origins [75].

Looking at the published literature on structural characteristics of *C. auris* clades, Kwon et al. [16] studied in detail a large number of Clade II isolates and reported their lower resistance to antifungals as compared to isolates of Clade IV. The latter clade was found to be resistant to multiple antifungal drugs, with some isolates being resistant to all three major antifungal classes (i.e., azoles, polyenes, and echinocandins) [76]. Microscopy observations and Raman analyses (given in Figure 1 and Figure 10, respectively) showed that both subclades of Clade II, despite being embedded in ECM, possess high amorphous fractions in their chitin structure as compared to Clade IV. On top of such an intrinsic weakness of the chitin structure, any drug-induced metabolic inhibition of chitin synthesis (i.e., the effect of aculeacin, echinocandin, and polyoxins) should promptly lead to lysis [77]. In a previous Raman study of *C. auris* exposed to Amphotericin B [76], a lack of metabolic reactiveness was noticed in LSEM643T (Clade II) as compared to LSEM3673 (Clade III) in counteracting the drug effect. Though a relative increase in chitin fraction was noticed in both clades, only Clade III was found capable of increasing chitin crystallinity and changing its polymorphic structure in an attempt to resist drug effects. The present study confirms that, although being quantitatively a minor cell wall component, chitin plays a fundamental role in inhibiting the action of antifungal disturbances to cell metabolism. Therefore, a genomic lack of trigger for structural changes of the cell wall is likely the main reason for the lower resistance to antifungals of Clade II isolates. Future studies could be designed to specifically address the relationship between Raman metabolomics and genomic analyses [78] in order to further elucidate the chemical origin(s) of the dissimilar stress resistances recorded among *C. auris* clades.

The cell walls of different *C. auris* clades/subclades also displayed different structures and complexities of glucan mannans (cf. parameter F_M_ in Figure 10). Though β-glucans and chitin are located in the innermost layer, mannans display in both EMC and the outer layer of the cell walls. Therefore, different mannan structures play a fundamental role in enhancing virulence and reducing immunogenic exposure in distinct ways. Upon checking the comparison again between LSEM3682 (Clade II) (embedded in EMC) and LSEM3662 (Clade IV) yeast cells, it can be seen that the former sample experiences the highest, and the latter the lowest, fraction of α–D–mannopyranose structure (cf. parameter F_M_ in Figure 10). The outer shell of fungal cell walls is highly dynamic as compared to the relatively rigid inner domain of chitin and glucans. However, though a consistent fraction of α–1,3–glucan is expected to be located in the stiff/hydrophobic core, the largest amount still stems from ECM and somewhat from the outer dynamic cell-wall domain. The fractions located at the environmental interface play a fundamental role in blocking immune recognition of β-glucan receptors in the host cells [79,80]. The α–1,3–glucan is indeed a very versatile molecule in the *Candida* cell structure; its high fractions, as found in both ECM and mannan structures of LSEM3682 (Clade II) cell walls, reveal the bi-functional strategy of this subclade: supporting cell walls through the formation of a strongly hydrophobic scaffold while increasing virulence by disabling host-cell detection.

Upon extending the comparison of the two somewhat antithetical structural characteristics/strategies of LSEM3682 (Clade II) and LSEM3662 (Clade IV) to the other clades investigated, one finds that the LSEM3690 (Clade I) lacks ECM but contains the highest fraction of α–D–mannopyranose, while also exploiting a relatively high fraction of core α–1,3–glucans. On the other hand, only LSEM3672 (Clade III) includes quite low contents in both core α–1,3–glucans and peripheral α–D–mannopyranose. In other words, though lacking ECM, this latter subclade is neither “buying” the immune-recognition blocking strategy of the LSEM3682 (Clade II), nor packing its α–1,3–glucans in the inner core of the cell walls to develop a strongly hydrophobic scaffold as in the cases of both Clade II subclades. Accordingly, the origin of high virulence/drug resistance of LSEM3672 (Clade III) should reside in a different metabolite strategy.

**Figure 13:**
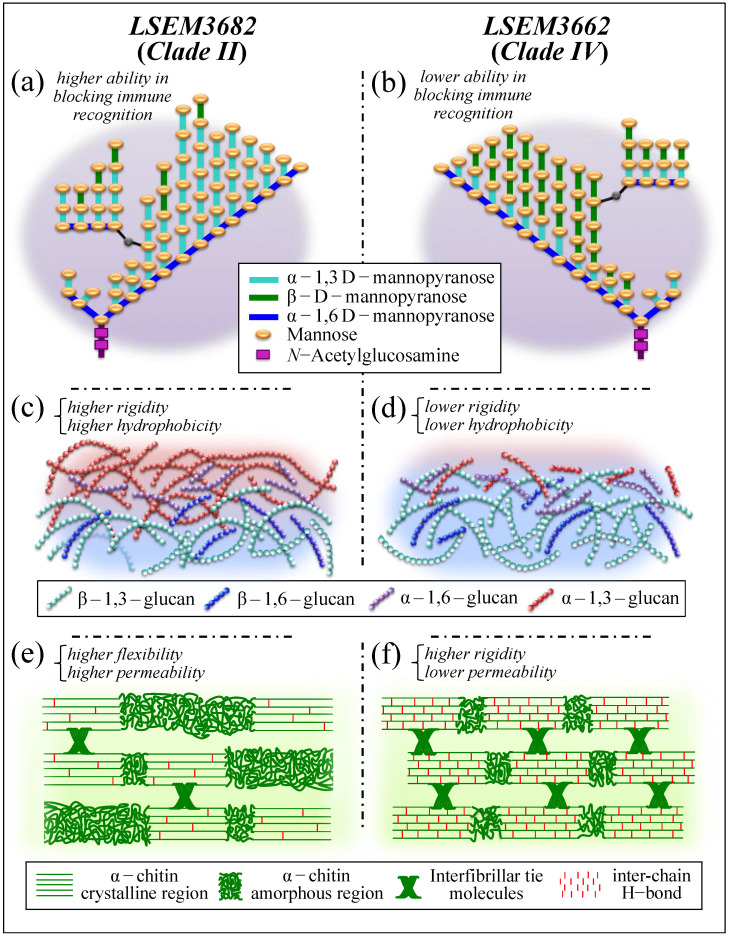
Summary, in three separate sections, of the structural differences in cell walls between LSEM3682 (Clade II) and LSEM3662 (Clade IV); (**a**,**b**): mannopyranose structure, (**c**,**d**): glucan structure, and (**e**,**f**): chitin structure.

Figure 13 summarizes in three separate sections the above-discussed structural differences in the cell walls of LSEM3682 (Clade II) and LSEM3662 (Clade IV): mannopyranose structure ((a) and (b)), glucan structure ((c) and (d)), and chitin structure ((e) and (f)).

As discussed above, on the basis of the Raman analysis of *Candida* metabolites in Figure 10, the sample LSEM3672 (Clade III) appears to possess the lowest amount of α–1,3–glucans, the second-lowest concentration of α–D–mannopyranose, and a relatively low chitin crystallinity. In other words, this clade apparently adopts none of the polysaccharide-related cell-wall strategies to ensure survivorship and proliferation. Despite being drug-resistant and highly virulent [8,81], its cell walls appear to possess low impermeability/hydrophobicity and scarce immune recognition blocking. What is, then, the strategy adopted by this quite “creative” subclade? The answer to this question should be sought in metabolomic characteristics different from polysaccharides.

As seen in Figure 10, LSEM3672 (Clade III) stands as the *C. auris* subclade incorporating the highest ergostane fraction. Ergostane-type steroids are chemical compounds characteristic of a variety of fungi, with a wide spectrum of biological activity also including immunological functions. Raman spectroscopy does not allow for judging the location of the detected ergostane molecules, namely, whether they are buried in the plasma membrane or cling to the outer dynamic cell-wall domain as glycosylated sterols. In either case, however, a key-role could be foreseen for the ergostane moiety. Modifications of the sterol composition of the plasma membrane could be interpreted as a strategy to stabilize the structure of the plasma membrane in order to obstruct the action of pore formation exerted by Amphotericin B [82]. On the other hand, the presence of ergostane molecules as glycosylated sterols in the outer layer of the cell walls could be construed as a ploy to dodge the pro-inflammatory mediators (i.e., nitric oxide, reactive oxygen species, and interleukin-1 beta) released by activated macrophages. In this latter context, Kikuchi et al. [83] reported on the inhibitory effect of a number of ergostane compounds on nitric oxide production by macrophages. If this interpretation is correct, the LSEM3672 (Clade III) should be classified as a subclade that selected a completely different path to virulence and whose structure underwent a quite unique evolution among all *C. auris* clades/subclades.

### 4.2. Raman Metabolomics: A Tool in Epidemiology of Invasive Candidiasis

Though databases of *Candida* genes are continuously updated, molecular techniques such as polymerase chain reactions are systematically applied to document the mechanisms of drug resistance arising from genetic mutations [84]. Despite its robustness, this combined approach is a technically demanding one and requires cumbersome elaborations on a case-by-case base to cover all resistant strains. As shown in the present study of pathogenic *C. auris* clades/subclades, Raman metabolomics has the potential to improve this situation by providing real-time speciation and key information about the structural variety of individual clades/subclades. Structural differences can be used as biomarkers to speciate the clades and to interpret the strategies followed by their specific isolates. Additional advantages given by Raman imaging are the possibility to monitor, in situ and in time lapse, drugs’ effectiveness, and to predict therapeutic outcomes. Raman speciation could boost modern methods of companion diagnostics [85], and in situ monitoring could expand the role of pharmacometabolomics as an emerging *modus operandi* in precision medicine [86,87]. The application of Raman spectroscopy to companion diagnostics of *Candida* infections could provide a new path for safely and effectively selecting drugs, giving healthcare professionals a robust platform to reduce patients’ risks and greatly lowering the chances of dangerous genetic mutations of the *Candida* pathogen. Time-lapse Raman imaging of selected *Candida* metabolite levels could provide the “reasons” for genomic mutations and enable monitoring the effects of drug compounds on specific metabolic pathways. Complementary to genomics, Raman-based pharmacodynamic approaches may provide insightful snapshots of cell metabolism and allow for visualizing the pathways and mechanisms involved in cell responses to different treatments. Raman metabolomics of *Candida* clades and subclades could also help to reproducibly assess observed interpatient variability by interpreting *Candida* metabolome diversities related to the physiological variables of different patients.

Escribano et al. [88] studied in detail the endemic genotypes of *Candida albicans* causing fungemia in a large number of patients from the same hospital. These researchers found that up to 25% of patients were infected by endemic *C. albicans* genotypes (more than half of them being located in the same ward of the hospital), thus demonstrating that the isolates might have originated from common sources, i.e., contact with healthcare workers, the use of catheters and other biomedical devices, parenteral nutrition, or other sources in the hospital environment. Despite the importance of these findings, the authors also pointed out the main limitation of their study as the failure to precisely determine the source(s) of infection and the route(s) of transmission in the hospital. The reason for such a failure should be tracked down to the extremely cumbersome amount of genomic analyses required to characterize isolates from the overall hospital environment, from health care workers, and from other anatomical sites of patients affected by candidemia. The availability of an agile, precise, and relatively inexpensive tool, such as Raman spectroscopic profiling of *Candida* isolates, could simplify and empower tracking procedures, thus greatly advancing the epidemiologic management of invasive candidiasis.

### 4.3. Future of Healthcare Management Using Raman Barcodes

As discussed in the previous section, the prompt administration of the appropriate antifungal drug(s) is key in the successful treatment of patients affected by *Candida* infections. The initial delivery of an appropriate antifungal drug should take place within the first 12 h after the first positive test of blood culture in order to maximize the cure outcome [89]. However, a prompt administration of the most effective drug is presently retarded by the slow turnover of the available susceptibility testing procedures. The quest for on-site real-time profiling of *Candida* infections represents just one specific aspect of a global trend aimed at accurately identifying diseases at their prodromal stage in order to avoid the acute stage and elongate the span of healthy living. Disease prevention and early diagnostics appear nowadays as the two main goals of global healthcare, with patients increasingly focused on self-care and quality of life and governments pinpointing reduced healthcare costs. In our modern times, several new tools have become available to make these goals possible; these include improved analytical techniques, algorithms of machine learning, and artificial intelligence approaches. The combination of these innovative tools could in the near future enable the collection and handling of a large volume of high-quality data in nearly real time, thus opening the way for new routes for high-efficiency healthcare management. 

In the present work, we have explored one possible high-efficiency route by demonstrating the possibility of speciating all four clades (and related subclades) of pathogenic *C. auris* by applying a “Raman barcode” approach built on machine-learning-construed spectral deconvolutions. Though the barcode technology is not new in modern healthcare, the method proposed here presents substantial differences with those presently in use. Barcodes are currently employed from manufacturing to dosing, in tracking batches of medicine, and in making distribution processes more efficient. In clinical settings, using barcodes has been shown to greatly increase accuracy in medication management [90,91]. The adoption of a Raman barcode could bring similar benefits, but it is conceptually different from the barcodes used thus far. It rigorously links to fundamental features of physical chemistry (i.e., the vibrational behavior of molecules), which in turn reflect cell metabolomics through a set of intentionally selected compositional parameters. A conceptually similar approach can be seen in the so-called “DNA barcode” [92,93]. DNA barcoding has successfully been used to identify viral and bacterial species through the analysis of specific regions of DNA in order to match with a reference library. However, the three main steps required for applying DNA barcoding, namely, DNA extraction, polymerase chain reaction amplification, and DNA sequencing and analysis, are exactly the same cumbersome elaborations hampering the on-site real-time profiling of *Candida* species. Therefore, the Raman barcode, which requires no or minimal sample preparation, might surpass the DNA barcode in swiftness and flexibility regarding on-site diagnostics.

As shown in this study, when Raman barcoding is applied to *Candida* cells, it gives indications on profiling and reveals metabolism-related parameters, which could be used in evaluating and predicting environmental stress adaptations and drug resistance. Raman barcodes, which unambiguously locate *C. auris* clades and subclades (cf. Figure 12), could be decrypted into easily readable information through appropriate apps including tracking information for a multiplicity of even slightly different isolates from patients. Once automatically linked to clouds, the Raman barcode could be developed into a capillary network for real-time decisions on appropriate antifungal drugs and management of pathogenic fungal infections.

## 5. Conclusions

A dual Raman approach has been applied to analyze the metabolites of four *C. auris* clades and related subclades for a total of seven different samples. First, representative Raman spectra were computed from averaging over 20 spectra collected at different locations on each sample with high spectral resolution and an optical probe including multiple cells in each acquired spectrum. Average spectra were deconvoluted according to a specifically tailored machine-learning algorithm linked to a previously built database of Raman spectra from elementary molecules. Then, several millions of spatially resolved spectra were taken with sub-micrometric spatial resolution in order to map the cell cultures. Maps were then converted into hyperspectral images of selected parameters for the purpose of metabolomic profiling. These two complementary Raman procedures gave consistent results, showing excellent reproducibility and thus the possibility of establishing a satisfactory level of standardization. The Raman analyses unveiled important differences in the way different clades/subclades are structured, with important implications for cellular morphogenesis, virulence, and drug resistance. This study demonstrates the importance of metabolomics as a parallel and complementary approach to genomics in profiling *C. auris* clades and subclades. Raman identification of *Candida* species down to the subclade level can be obtained in a span of minutes, thus reducing the time necessary for optimizing antifungal treatment decisions to a minimum.

In conclusion, Raman metabolomics empowered by machine-learning algorithms and barcode technology, as presented in this paper, could become a powerful tool in efficiently profiling and tracking *Candida* infections down to the sub-clade level. The Raman approach could operate hand-in-hand with other global health trends, such as enabling companion diagnostics, point-of-care testing, and remote healthcare. Personalized medicine carried out through on-site real-time Raman analyses, decentralization of healthcare exploiting relatively inexpensive Raman devices, and acquisition of cloud-based big data through applying Raman barcodes could represent a new path toward the medicine of the future.

## Data Availability

Data are available upon reasonable request.
